# Selenium Treatment and Chagasic Cardiopathy (STCC): study protocol for a double-blind randomized controlled trial

**DOI:** 10.1186/1745-6215-15-388

**Published:** 2014-10-06

**Authors:** Pedro Emmanuel Alvarenga Americano do Brasil, Andréa Pereira de Souza, Alejandro Marcel Hasslocher-Moreno, Sérgio Salles Xavier, Sonia Regina Lambert Passos, Maria de Fátima Ramos Moreira, Marília Santini de Oliveira, Gilberto Marcelo Sperandio da Silva, Roberto Magalhães Saraiva, Claudia Santos de Aguiar Cardoso, Andréa Silvestre de Sousa, Mauro Felippe Felix Mediano, Maria da Gloria Bonecini de Almeida, Otacílio da Cruz Moreira, Constança Britto, Tania Cremonini de Araújo-Jorge

**Affiliations:** Chagas Disease Clinical Research Laboratory, Evandro Chagas National Institute of Infectious Diseases, Oswaldo Cruz Foundation, Avenida Brasil 4365, Manguinhos, Rio de Janeiro, 21040-360 Brazil; Laboratory for Innovations in Therapy, Teaching and Bioproducts, Oswaldo Cruz Foundation, Avenida Brasil 4365, Manguinhos, Pavilhão Cardoso Fontes, Sala 64, Rio de Janeiro, 21040-360 Brazil; Laboratory of Epidemiology, Evandro Chagas National Institute of Infectious Diseases, Oswaldo Cruz Foundation, Avenida Brasil 4365, Manguinhos, Rio de Janeiro, 21040-360 Brazil; Laboratory of Toxicology, Metal Sector, Center for the Study of Workers’ Health and Human Ecology (CESTEH), National School of Public Health Sergio Arouca (ENSP), Oswaldo Cruz Foundation, Avenida Brasil 4365, Manguinhos, Rio de Janeiro, 21040-360 Brazil; STD/AIDS Clinical Research Laboratory, Evandro Chagas National Institute of Infectious Diseases, Oswaldo Cruz Foundation, Avenida Brasil 4365, Manguinhos, Rio de Janeiro, 21040-360 Brazil; Nutrition Service, Evandro Chagas Hospital, Evandro Chagas National Institute of Infectious Diseases, Oswaldo Cruz Foundation, Avenida Brasil 4365, Manguinhos, Rio de Janeiro, 21040-360 Brazil; Laboratory of Immunology and Immunogenetics, Evandro Chagas National Institute of Infectious Diseases, Oswaldo Cruz Foundation, Avenida Brasil 4365, Manguinhos, Rio de Janeiro, 21040-360 Brazil; Laboratory of Endemic Diseases and Molecular Biology, Evandro Chagas National Institute of Infectious Diseases, Oswaldo Cruz Foundation, Avenida Brasil 4365, Manguinhos, Rio de Janeiro, 21040-360 Brazil

**Keywords:** Chagas cardiomyopathy, Chagas disease, Clinical Trial, Selenium, *Trypanosoma cruzi*

## Abstract

**Background:**

Heart disease progression occurs in 30% of patients with chronic *Trypanosoma cruzi* infection. Supplementation with selenium (Se) in animal model of *T. cruzi* infection produced promising results. There is evidence that patients with Chagas heart disease have lower Se levels than healthy individuals and patients with *T. cruzi* infection without of cardiac disease. The aim of this investigation is to estimate the effect of Se treatment on prevention of heart disease progression in patients with chagasic cardiopathy.

**Methods:**

The Selenium Treatment and Chagasic Cardiopathy trial is a superiority, double-blind, placebo-controlled, randomized clinical trial. The eligibility criteria are as follows: (1) a Chagas disease diagnosis confirmed by serology; (2) segmental, mild or moderate global left ventricular systolic dysfunction; and (3) age between 18 and 65 years. The exclusion criteria are as follows: (1) pregnancy, (2) diabetes mellitus, (3) tobacco use, (4) alcohol abuse, (5) evidence of nonchagasic heart disease, (6) depression, (7) dysphagia with evidence of food residues in the esophagus, (8) dysphagia with weight loss higher than 15% of usual weight in the last four months and/or (9) conditions that may result in low protocol adherence. The intervention will be 100 μg of sodium selenite once daily for 365 consecutive days compared to placebo. The following are the primary outcomes to be measured: (1) the trajectories of the left ventricular ejection fraction in the follow-up period; (2) reduction of heart disease progression rates, with progression defined as a 10% decrease in left ventricular ejection fraction; and (3) rate of hospital admissions attributable to dysrhythmia, heart failure or stroke due to Chagas disease. One hundred thirty patients will be randomly allocated into either the intervention or placebo group at a ratio of 1:1. The sequence allocation concealment and blinding were planned to be conducted with the strategy of numbered boxes. Both patients and health-care providers will remain blinded to the intervention groups during the 5 years of follow-up.

**Discussion:**

If Se treatment reduces the progression of Chagas cardiopathy, the inclusion of this micronutrient in the daily diet can improve the therapeutic regimen for this neglected tropical disease at low cost.

**Trial registration:**

Clinical Trials.gov ID: NCT00875173 (registered 20 October 20 2008).

## Background

Chagasic cardiopathy is considered the most common and serious manifestation of chronic Chagas disease [1]. This specific form of Chagas disease is associated with high levels of morbidity and mortality [2] and is also a major component in Chagas disease burden [3].

The probability of death among patients with heart failure (HF) attributable to Chagas disease is about 80% over the course of 10 years [4]. According to the Brazilian consensus on Chagas disease [2], patients with *Trypanosoma cruzi* infection and heart disease can be classified into different stages, depending on the electrocardiography (ECG) and echocardiography findings, as follows: stage A (abnormalities on ECG attributable to Chagas disease and no abnormalities detected by echocardiography), stage B1 (abnormal echocardiogram showing ejection fraction (EF) >45% and no HF syndrome), stage B2 (echocardiogram showing EF <45% and no HF), stage C (HF with improvement after treatment optimization) and stage D (HF with no improvement after treatment optimization) [2]. This classification is closely related to prognosis, as left ventricular (LV) function is the strongest predictor of mortality in Chagas heart disease [4–6].

Selenium (Se) is a vital trace element for all organisms, and it has been considered the most important antioxidant mineral, being essential to selenoenzymes such as glutathione-peroxidase (GPx) and others [7]. Se levels vary among the foods of different countries, and a serum level lower than 45 μg/L is considered a potential risk factor for heart disease [8]. The plateau of maximal Se-GPx activity is reached when the plasma Se concentration ranges from 89 to 114 μg/L [8]. Se function is related to its localization in active sites in proteins, as well as to how these proteins, such as GPx, are involved in the protection of biomembranes against the attack of free radicals [9].

Some diseases have been correlated with low serum levels of Se, including AIDS [10], Kashin–Beck disease [11], myxedematous cretinism [12], prostate cancer [13] and cardiopathies [8]. In some cases, low Se levels may be an effect of particular diseases or may contribute to exacerbation of disease progression.

Nutritional Se deficiency or a low Se level is associated with some cardiopathies in animals and humans. There is strong evidence that prolonged Se deficiency in patients receiving home parenteral nutrition is harmful to the heart and can contribute to the development of myocardiopathy [14, 15]. Keshan disease is among the cardiopathies that are classically related to Se deficiency. This condition affects children and young women of childbearing age in areas of China and Eastern Siberia with low soil Se levels [16, 17]. In addition, it is possible that low Se could contribute to the development of AIDS-related myocardiopathy [18]. Similar evidence came from autopsic studies of AIDS patients with heart disease and decreased Se levels [19]. Low Se levels and hyperprolactinemia were reported in patients with peripartum cardiopathy in Africa’s Sahel region, an uncommon form of congestive myocardiopathy that occurs in the last month of pregnancy or in the first 6 months postpartum [20]. Also, Se deficiency may be a contributing factor in congestive HF that affects malnourished children with kwashiorkor disease [21]. There is a positive correlation between Se intake and its blood levels [22], and it has been suggested that Se may play a role in the clinical severity of HF, as evidence indicates that mean Se intake is lower in these patients. Ischemic heart disease has also been linked to low Se levels [23–25].

There is only a single study in which investigators evaluated the relationship between Se levels and chagasic cardiopathy in humans [26]. Nevertheless, there is some consistent evidence showing that low Se levels worsen cardiopathy in Chagas disease animal models [27, 28] and that Se supplementation is beneficial in Chagas disease cardiopathy animal models [29–31]. The mechanisms involved in this effect are modulation of tissue inflammation and oxidative stress [32, 33].

The occasional ingestion of an elevated dose of Se can cause signs of toxicity. Such effects may be seen in association with suicide attempts and accidental ingestion [34]. However, adverse effects seem to be rare and have been reported only in cases of exposure to Se doses over 400 μg/day [14]. Acute symptoms include irritability, pain and trembling; tachycardia; nausea, vomiting and abdominal pain [34–36]; elevated levels of bilirubin and alkaline phosphatase; and altered levels of thyroid hormones [37]. Severe acute and chronic symptoms include pulmonary edema and lesions [36, 38] and garlic breath and diarrhea [34, 35]. Chronic exposure to high Se doses can lead to longer prothrombin time (a marker of hepatic injury); alopecia or dry hair and nails; and convulsions, paralysis and hemiplegia [39–41]. Possible treatments for patients with a diagnosis of acute Se poisoning are gastric wash and induction of vomiting by use of emetics [34]. Monitoring is recommended in mild cases (that is, reactions that do not interfere with activities of daily living), and Se intake may be indefinitely or temporarily interrupted until the symptoms completely disappear in patients with moderate or severe adverse reactions.

### Main objective

**Figure 1:**
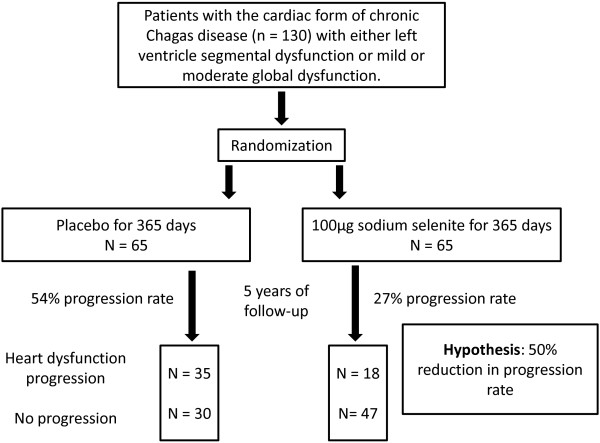
**Randomization, hypothesis and primary end point.**

Our hypothesis in the present study is that Se supplementation will act as an immune modulator, reducing the inflammation and injures in the heart over time. Our aim is to estimate the effects of Se intake on Chagas heart disease progression, expressed as the decline in left ventricular ejection fraction (LVEF) (Figure 1).

### Specific objectives

The following are our specific objectives:

1. To compare the trajectories of LVEF in both intervention groups.
2. To estimate and compare the disease progression incidence rates in both intervention groups.
3. To compare the LVEF average of both intervention groups at the end of the follow-up period.
4. To estimate the effect of Se intake on quality of life.
5. To estimate the Se intake safety level.
6. To follow Se serum levels during the period of Se prescription and possible relation to Se toxicity.
7. To describe nutritional status and Se feeding habits.

## Methods/design

### Ethical considerations

The trial was approved by the Evandro Chagas National Institute of Infectious Diseases (INI) Research Ethics Committee (CEP-INI, CAAE 0043.0.009.000-04) and conforms to standards currently applied by the Brazilian National Committee for Research Ethics (CONEP). An external data safety monitoring board (DSMB) will monitor this trial to guarantee the quality of the study. The trial protocol may be altered, with confirmation from the DSMB, if interim analyses demonstrate statistically significant differences in the primary end point between groups. If significant beneficial effects of Se supplementation are found, Se treatment will be offered to those patients who were randomized to the placebo group.

### Study design and settings

This is a single-center, double-blind, placebo-controlled, superiority randomized clinical trial. Individuals followed at INI will be recruited to participate in the study. INI is a national reference center for treatment and research in infectious diseases and tropical medicine in Brazil. The majority of patients with suspected Chagas disease in Rio de Janeiro State are referred to the INI outpatient unit, either for diagnostic investigation or for specialized health care. At the end of 2012, about 1,100 patients with Chagas disease were followed in an outpatient unit at INI. This unit is able to proceed with more than 100 medical appointments per week for specialized health care for Chagas disease patients. The unit staff is made up of infectious disease specialists, cardiologists, gastroenterologists, nurses, pharmacists and exercise physiologists. Resources such as echocardiography, tomography, 24-hour Holter ECG monitoring, digestive endoscopy and cardiac rehabilitation are also available. Patients receiving health care at this unit are mostly migrants from rural areas who are currently living in the Rio de Janeiro metropolitan area.

### Participants

Patients will be recruited sequentially during their routine outpatient visits. The eligibility criteria are as follows:

1. A confirmed Chagas disease serological diagnosis according to the Brazilian consensus diagnostic investigation recommendation [2].
2. Segmental mild or moderate global left ventricular systolic dysfunction.
3. Age between 18 and 65 years.

The following are the exclusion criteria:

1. Pregnancy or breastfeeding
2. Diabetes mellitus
3. Tobacco use
4. Alcohol abuse
5. Evidence of nonchagasic heart disease
6. Depression
7. Dysphagia with evidence of food residues in the esophagus
8. Dysphagia with weight loss higher than 15% of usual weight in previous four months
9. Medical prescription of vitamins or supplements
10. Residence close to mineral deposits, metal industries or places with radioactive exposure
11. Participation as a volunteer in other clinical investigations with interventions
12. Conditions that may result in low protocol adherence.

Patients who meet the inclusion criteria will be invited to participate in the study, and a signed written informed consent will be obtained from those patients willing to participate. Participants will undergo an initial interview and baseline blood and urine tests. Initial interviews and tests will include a questionnaire to collect demographic data, medical history, quality of life and detailed information for a nutritional profile including diet *Se* intake, clinical and cardiovascular examinations (ECG and echocardiography) and biochemical and hematological laboratory measurements.

### Intervention

Patients will receive either 100-μg sodium selenite capsules or placebo capsules to be taken orally after a meal once daily for 365 days. The placebo capsules will have the same color, form and texture as those containing Se. At each visit, patients in both groups will receive the number of capsules to be taken until the next scheduled appointment. If, for any reason, treatment interruption is necessary, then the treatment time will be extended until the volunteer takes 365 capsules.

Sodium selenite is not commercialized in isolated form, but is available in combination with other vitamins and supplements. Therefore, a partnership was made with Relthy Technology in Pharmaceutical Services (Indaiatuba, Brazil). That company will be responsible for providing the capsules of sodium selenite and placebo for this investigation.

### Outcomes

There are two main outcomes that will be evaluated. (1) The LVEF will be treated as a continuous measure over time; therefore, the trajectories of both intervention groups will be compared. (2) Disease progression as a main outcome measure will be defined as a 10% decrease in the LVEF, death attributable to Chagas heart disease or hospital admission due to dysrhythmia, stroke or HF attributable to Chagas heart disease.

Four secondary outcomes will be assessed. (1) Disease progression as a secondary outcome measure will be defined as new findings attributable to Chagas heart disease, as compared to the previous tests, based on ECGs (such as intraventricular and atrioventricular node conduction disturbances, electrically inactive areas, T-wave abnormalities, supraventricular and ventricular arrhythmias, bradyarrhythmias, right bundle branch block and left anterior hemiblock or other findings attributable to Chagas disease) or echocardiograms (such as segmental dyskinesia, hypokinesia or akinesia, ventricular aneurysms, global dysfunction or other findings attributable to Chagas disease). (2) Quality of life will be measured with the brief version of the World Health Organization Quality of Life (WHOQOL-Brief) instrument [42]. (3) Reversion rates will be based on comparison of ECG or echocardiogram alterations from previously observations. (4) Adverse events will be recorded.

Volunteers will be evaluated for 5 years after baseline. The outcomes will be blindly assessed every 6 months in the first year. Echocardiography will be conducted every year from the second to fifth years of follow-up. ECG, WHOQOL and other clinical outcomes will be assessed every 6 months from the first to fifth years of follow-up. Follow-up visits to check for adverse events are planned to occur every 3 weeks for the first 2 months, every 2 months for the first year and every 6 months from the second to fifth years of follow-up.

Echocardiographic imaging will be conducted with a Vivid 7 ultrasound system (GE Medical Systems, Milwaukee, WI, USA) equipped with a four-matrix transducer from 2 to 4 MHz. All examination results will be recorded and stored in digital media to allow later analysis at an EchoPAC PC workstation with software version 108.1.12 (GE Medical Systems). The images will be evaluated with the standard left ventricle windows: long and short left parasternal axes (basal, middle and apical) and apical three, four and two chambers. All these images will be recorded with myocardial movements and ECG tracing. Cardiac dimensions will be measured according to the recommendations of the European Society of Echocardiography [43]. LVEF and systolic and diastolic LV final volumes will be estimated according to Simpson’s rule. Segmental analysis will be conducted with the standard 17 segments description. ECGs will be recorded with the standard 12-lead protocol while the patient is at rest. Digital images of the test will be stored in a workstation and printed out on graph paper for later analysis.

Quality of life will be assessed with the WHOQOL-Brief instrument, which was previously validated in a Brazilian population [42]. It is a generic instrument that is easy to understand and apply. It consists of 26 questions in 4 dimensions: physical, psychological, social relationships and environment.

### Sample size

We estimated a minimum sample size of 130 volunteers (65 in the placebo group and 65 in the Se group). In this estimate calculation, we considered an α error of 0.05, a β error of 0.20 and a difference in progression risk of 50%. The calculation assumed a progression rate scenario for cardiopathy based on findings for LVEF decline in a previous cohort study conducted in Brazil (Virgem da Lapa, State of Minas Gerais) [44]. See Table 1 for details.

**Table 1:** **Minimum sample size estimation** ^**a**^

Parameters	Groups	
	Placebo (***n***= 65)	Selenium (***n***= 65)
Progression rate	54%	27%
Confidence level	95%	95%
Proportion	1:1	1:1

^a^Estimate was calculated with an α error of 0.05, a β error of 0.20 and progression rates in 5 years.

### Randomization

A sequence was computer-generated to randomly allocate 130 patients into 2 groups in a 1:1 ratio. This sequence was generated in blocks of 4, 8 and 12 using the “blockrand” extension of the R Project software package. This sequence is available only to a pharmacist not involved in volunteer recruitment. This pharmacist decided which group would be the placebo group and which would be the intervention group by flipping a coin. Next, opaque boxes will be filled either with placebo or sodium selenite capsules and later sealed and numbered to correspond to the computer-generated sequence. As patients undergo volunteer recruitment by the medical staff, they will be assigned a number sequentially corresponding to a treatment box. Therefore, a strategy of numbered boxes will be used for sequence concealment.

### Blinding

Patients, health-care providers and staff involved in outcome assessment were blinded to treatment. The blinding was conducted using the same strategy of allocation concealment by numbered boxes. Therefore, numbers were assigned to volunteers’ treatments, but only one pharmacist not involved in these tasks will be aware of what is in each numbered box.

### Statistical analysis

The type of analysis of major interest is the intention-to-treat analysis. At baseline, both intervention groups will be compared concerning information that might modify disease prognosis to check if random allocation worked properly at that time. The main analysis will be longitudinal modeling of LVEF by comparing the two intervention groups. This modeling will be conducted with marginal models and conditional models. This analytical strategy will also be used for other continuous outcomes, such as the WHOQOL-Brief.

For each binary outcome of interest, incidence rates will be estimated and compared among the different intervention groups, including the adverse events discriminated by severity and causality. Time-dependent survival models will be adjusted for the binary outcomes, as progression may occur more than once for each participant. The survival modeling extension for multiple events of Prentice, Williams and Peterson will be adopted [45].

Secondary analysis will be conducted as per protocol by adjusted regression. This secondary analysis will be conducted mainly if there is an understanding that random allocation did not fulfill its purposes at baseline.

REDCap software [46] will be used for data management, and data analysis will be conducted with R Project software [47].

### Interim analyses and stopping rules

Four interim analyses are planned. The first will be conducted when the last volunteer completes 6 months of follow-up, the second will be conducted when the last volunteer completes 18 months of follow-up, the third will be conducted when the last volunteer completes 30 months of follow-up and the last will be conducted when the last volunteer completes 42 months of follow-up. Every interim analysis will be submitted for DSMB approval.

Trial interruption for ethical reasons may be recommended by the DSMB and confirmed by the trial coordinators or by the trial coordinators and confirmed by the DSMB. Trial interruption may be recommended due to either positive or negative results exceeding expectations. The prespecified stopping rule is a marginal 10% difference in LVEF among groups, a reduction of progression rate in 50% or more participants observed at the main outcome measurement points, an increase of 10% or more of severe adverse events in one of the intervention groups or serious adverse events in at least 5% of the volunteers. All these estimates should have a significance level of 0.01 or less in any of the interim analyses.

## Discussion

This clinical trial presents a possible new therapy based on the regulation of inflammation and fibrosis with the potential of delaying the progression of cardiopathy in patients with chronic Chagas heart disease. If the hypothesis is confirmed, it will constitute a new contribution to the improvement of the health care of the affected population, with prospective application in all of Latin America, where the WHO registers the existence of about 10 million infected people [48], and 30% of them (3 million to 4 million people) are expected to develop the cardiac form. Supplementation with micronutrients is simple and has been widely employed by oral administration [49]. In many countries, including the United States and France, Se is considered a supplement instead of a medicine. According to the Brazilian Agency of Sanitary Inspection, 100 μg of Se is considered medicinal because it is up to 100% of the recommended 34-μg daily intake. However, the daily level in our trial is within the limits considered safe (150 μg maximum; Secretariat of Health Surveillance, Ministry of Health (SVS/MS) Ordinance 40/1998). The inorganic form of Se (sodium selenite) was chosen because it has been shown to prevent Keshan cardiopathy, to reduce electroechocardiographic alterations in patients nourished by the parenteral route and to reduce reinfarction and cardiac death due to acute myocardial infarction [50–52]. Because Chagas heart disease progresses slowly (1% to 3% per year) [5], this clinical trial will last 5 years. This duration is short compared to the natural progression rate of this disease. If Se treatment turns out to be beneficial in this trial, a new and affordable treatment strategy for Chagas heart disease will be readily available for people with chronic *T. cruzi* infection all over the world.

### Trial status

Participants are currently being recruited.

## Abbreviations

DSMB: Data safety monitoring board
ECG: Electrocardiography
EF: Ejection fraction
GPx: Glutathione peroxidase
HF: Heart failure
INI: Evandro Chagas National Institute of Infectious Diseases
LV: Left ventricle
LVEF: Left ventricular ejection fraction
STCC: Selenium Treatment and Chagasic Cardiopathy
Se: Selenium
WHO: World Health Organization
WHOQOL: World Health Organization Quality of Life instrument.
